# 8-Chloro-Cyclic AMP and Protein Kinase A I-Selective Cyclic AMP Analogs Inhibit Cancer Cell Growth through Different Mechanisms

**DOI:** 10.1371/journal.pone.0020785

**Published:** 2011-06-10

**Authors:** Simona Lucchi, Davide Calebiro, Tiziana de Filippis, Elisa S. Grassi, Maria Orietta Borghi, Luca Persani

**Affiliations:** 1 Laboratory of Endocrine and Metabolic Research, Istituto Auxologico Italiano, Milan, Italy; 2 Dipartimento di Scienze Mediche, Università degli Studi di Milano, Milan, Italy; 3 Laboratory of Immunology, Istituto Auxologico Italiano, Milan, Italy; 4 Dipartimento di Medicina Interna, Università degli Studi di Milano, Milan, Italy; Mayo Clinic, United States of America

## Abstract

Cyclic AMP (cAMP) inhibits the proliferation of several tumor cells. We previously reported an antiproliferative effect of PKA I-selective cAMP analogs (8-PIP-cAMP and 8-HA-cAMP) on two human cancer cell lines of different origin. 8-Cl-cAMP, another cAMP analog with known antiproliferative properties, has been investigated as a potential anticancer drug. Here, we compared the antiproliferative effect of 8-Cl-cAMP and the PKA I-selective cAMP analogs in three human cancer cell lines (ARO, NPA and WRO). 8-Cl-cAMP and the PKA I-selective cAMP analogs had similarly potent antiproliferative effects on the BRAF-positive ARO and NPA cells, but not on the BRAF-negative WRO cells, in which only 8-Cl-cAMP consistently inhibited cell growth. While treatment with the PKA I-selective cAMP analogs was associated with growth arrest, 8-Cl-cAMP induced apoptosis. To further investigate the actions of 8-Cl-cAMP and the PKA I-selective cAMP analogs, we analyzed their effects on signaling pathways involved in cell proliferation and apoptosis. Interestingly, the PKA I-selective cAMP analogs, but not 8-Cl-cAMP, inhibited ERK phosphorylation, whereas 8-Cl-cAMP alone induced a progressive phosphorylation of the p38 mitogen-activated protein kinase (MAPK), via activation of AMPK by its metabolite 8-Cl-adenosine. Importantly, the pro-apoptotic effect of 8-Cl-cAMP could be largely prevented by pharmacological inhibition of the p38 MAPK. Altogether, these data suggest that 8-Cl-cAMP and the PKA I-selective cAMP analogs, though of comparable antiproliferative potency, act through different mechanisms. PKA I-selective cAMP analogs induce growth arrest in cells carrying the BRAF oncogene, whereas 8-Cl-cAMP induce apoptosis, apparently through activation of the p38 MAPK pathway.

## Introduction

Cyclic AMP (cAMP) is an ancient and ubiquitous chemical messenger, being found both in prokaryotes and eukaryotes. In vertebrates it is a major intracellular mediator of neurotransmitters and hormones and regulates essential cell functions, such as contraction, secretion and replication. While cAMP has an antiproliferative effect on most cell types, it provides an opposite, i.e. pro-mitotic, stimulus for neurons and several cells of endocrine origin [1], [2]. Not surprisingly then, genes encoding key elements of the cAMP pathway act as oncogenes or oncosuppressors, most exquisitely in endocrine cells [3], [4], [5], [6], [7], [8], [9], [10]. Moreover, an upregulation of type I isoforms of the cAMP-dependent protein kinase A (PKA) has been documented in several malignancies [11].

Since cAMP has an antiproliferative effect on tumor cells, cell-permeable cAMP analogs have been considered for the therapy of human cancer [11]. 8-Cl-cAMP, the best studied of these compounds, has antiproliferative properties both *in vitro* and *in vivo* and has been evaluated in phase I/II clinical trials [11], [12], [13]. Yet, despite the well-documented effects of 8-Cl-cAMP, there is no common agreement on its mechanism of action. In the pioneering studies by the group of Yoon Cho-Chung it was in fact shown that 8-Cl-cAMP modifies the ratio of the PKA regulatory (R) subunits (type I vs. type II) by decreasing the levels of type I R subunits [11], [14]. Though this phenomenon was deemed responsible for the antiproliferative effect of 8-Cl-cAMP, the results of more recent studies suggest that the effects of 8-Cl-cAMP are instead mediated by its metabolite 8-Cl-adenosine and are independent of PKA activation and/or alterations of the ratio between type I and type II R subunits [15], [16], [17], [18].

In a previous work we found that a pair of site-specific cAMP analogs (8-PIP-cAMP and 8-HA-cAMP), which, when used in combination, selectively activate PKA I, had a potent antiproliferative effect on two BRAF-positive carcinoma cell lines (ARO and NPA), but not on the BRAF-negative WRO cells [19]. In this study we compared the effects of 8-Cl-cAMP and these PKA I-selective cAMP analogs on the same carcinoma cell lines (ARO, NPA and WRO), by looking at parameters such as cell growth, apoptosis and modifications of key signaling cascades that might be implicated in their antiproliferative effects.

## Results

### Effect of 8-Cl-cAMP or the PKA I-selective cAMP analogs on cell proliferation

**Figure 1:**
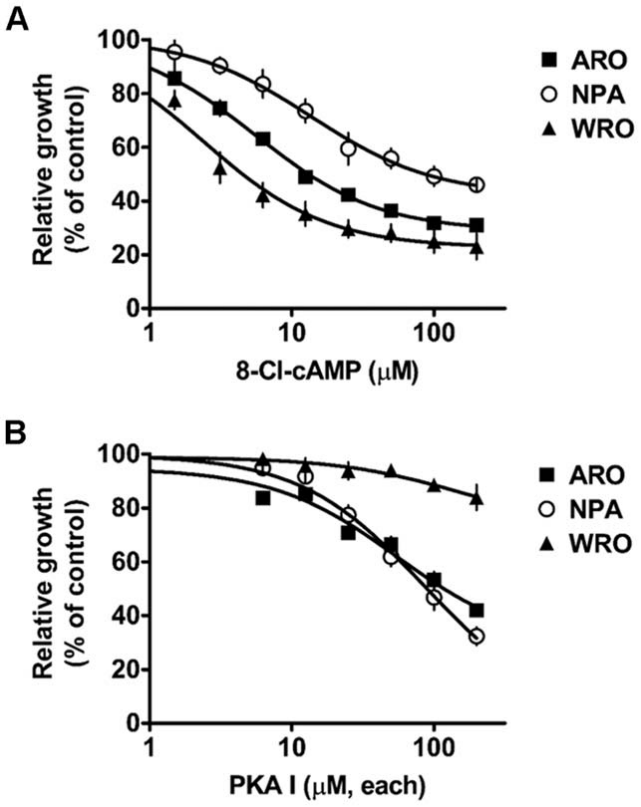
Antiproliferative effect of 8-Cl-cAMP (A) or the PKA I-selective cAMP analogs (B). ARO, NPA and WRO cells were cultured for 72 h in the presence of the indicated concentrations of either type of cAMP analog(s) and cell viability was determined utilizing the MTT assay. PKA I, PKA I-selective analogs used at equimolar concentrations.

First, we compared the antiproliferative effect of 8-Cl-cAMP and the PKA I-selective cAMP analogs. For this purpose, we treated ARO (colon cancer), NPA (melanoma) and WRO (follicular thyroid carcinoma) cells with different concentrations of 8-Cl-cAMP or the PKA I-selective cAMP analogs for various periods of time (24–96 h) and evaluated cell viability utilizing the MTT assay. The results indicated that both treatments were similarly potent in inhibiting the growth of ARO and NPA cells, whereas only 8-Cl-cAMP had a consistent antiproliferative effect on WRO cells (Figure 1). The effect of both treatments reached a maximum after 72–96 h of incubation (data not shown) and was dose-dependent, with IC50 values of 55.3 µM in ARO and 84.8 µM in NPA cells for the PKA I-selective cAMP analogs and between 2.3 and 13.6 µM for 8-Cl-cAMP in all three cell lines. Consistent with the previous finding that the antiproliferative effect of 8-Cl-cAMP is PKA-independent and at least partially mediated by its metabolite 8-Cl-adenosine [15], [16], [17], [18], the effect of 8-Cl-cAMP was significantly inhibited by treatment with the phosphodiesterase inhibitor IBMX, which blocks the conversion of 8-Cl-cAMP to 8-Cl-adenosine. On the contrary, IBMX did not modify the antiproliferative effect of the PKA I-selective cAMP analogs (Figure S1). Furthermore, the antiproliferative effect of 8-Cl-cAMP did not appear to be mediated by PKC, as treatment with a PKC inhibitor did not modify the response to 8-Cl-cAMP (Figure S2).

### Effect of 8-Cl-cAMP or the PKA I-selective cAMP analogs on cell cycle progression and apoptosis

Subsequently, we evaluated whether the antiproliferative effect of 8-Cl-cAMP and the PKA I-selective cAMP analogs was associated with growth arrest and/or apoptosis. This issue was addressed by a combination of approaches.

First, we analyzed the effects of 8-Cl-cAMP and the PKA I-selective cAMP analogs on cell cycle and apoptosis by flow cytometry analysis of the nuclear DNA content. For this purpose, ARO, NPA and WRO cells were treated with either type of cAMP analog(s) for different periods of time and analyzed after DNA staining with propidium iodide. 8-Cl-cAMP treatment was associated with a reduction of cells in G0/G1 and an accumulation of cells in S phase. In addition, it was accompanied by a significant increase in the fraction of apoptotic cells, i.e. those with a sub-G0/G1 DNA content. By contrast, exposure to the PKA I-selective cAMP analogs did not cause a significant increase in apoptosis. The only appreciable effect of the PKA I-selective cAMP analogs was a slight increase in the number of cells in G0/G1 (statistically significant in ARO cells) and a tendency towards a reduction of the cells in S and G2/M phases, both compatible with accumulation in G0/G1 (Table 1).

**Table 1:** Effects of 8-Cl-cAMP and the PKA I-selective analogs on cell cycle.

		Cell cycle phase (% of cells)			
Cell line	Treatment	Sub-G0/G1	G0/G1	S	G2/M
**ARO**	**control**	1.04±0.19	49.37±5.56	16.25±3.4	32.62±6.99
	**8-Cl-cAMP**	5.16±0.24‡	38.88±2.69*	22.10±2.79*	26.88±4.21
	**PKA I**	1.11±0.20	59.03±4.59*	12.52±1.10	24.29±4.26
**NPA**	**control**	1.81±0.15	57.21±2.06	20.56±1.44	19.6±1.17
	**8-Cl-cAMP**	9.25±2.08†	40.81±1.38†	27.37±1.52‡	21.59±1.86
	**PKA I**	2.88±0.69	61.34±1.73	17.81±1.24	17.19±1.26
**WRO**	**control**	3.30±0.25	50.11±0.69	16.06±1.79	30.94±1.77
	**8-Cl-cAMP**	17.63±1.35†	27.34±2.36†	23.60±1.75‡	31.81±2.21
	**PKA I**	6.165±0.50	54.80±3.35	15.28±1.71	23.99±4.90

Cells were treated with 8-Cl-cAMP (100 µM) or the PKA I-selective cAMP analogs (100 µM each) for 72 h. PKA I, PKA I-selective cAMP analogs. *, P<0.05 vs. control. ‡, P<0.01 vs. control. †, P<0.001 vs. control.

**Figure 2:**
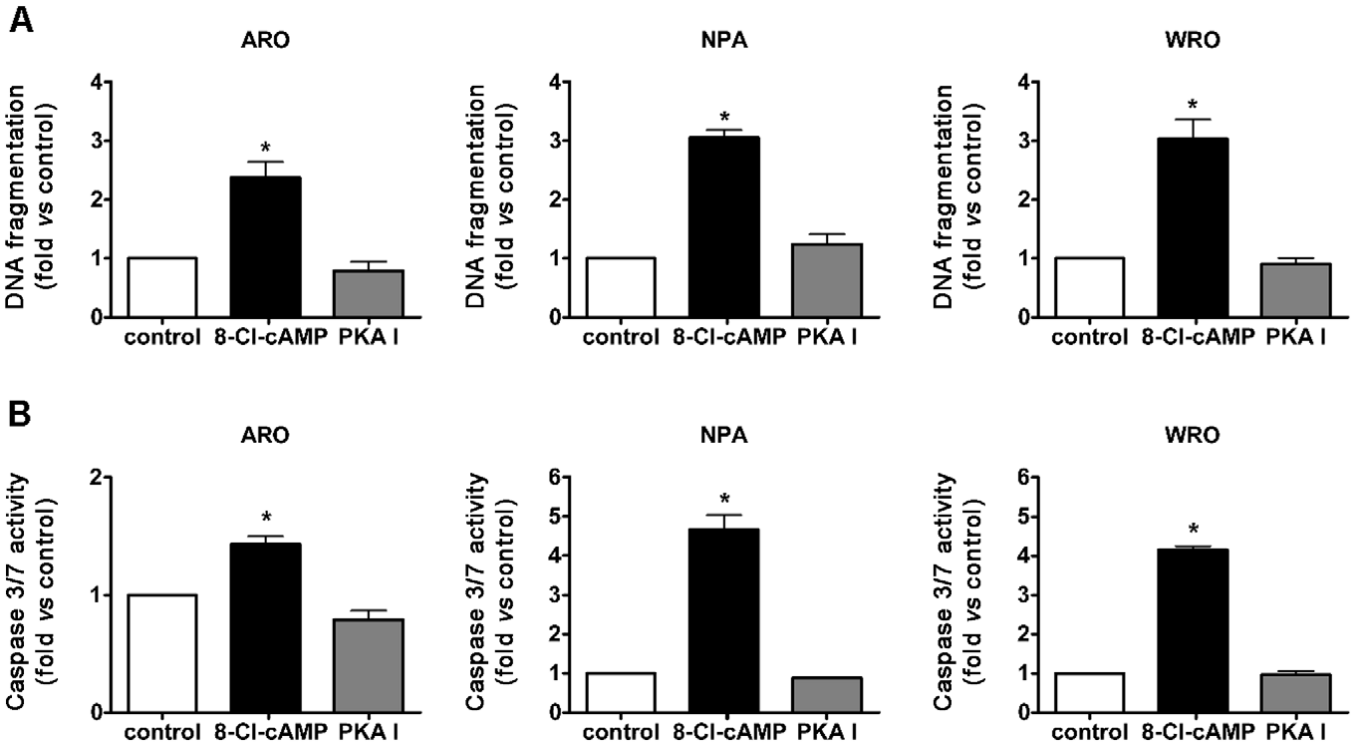
Effect of 8-Cl-cAMP or PKA I-selective cAMP analogs on DNA fragmentation and caspase 3/7 activation. A: effect on DNA fragmentation. Cells were exposed to 8-Cl-cAMP (100 µM) or the PKA I-selective cAMP analogs (100 µM each) for 48 h and the release of histone-bound DNA fragments into the cytosol was evaluated by a specific ELISA. Data are from three independent experiments. B: effect on caspase 3/7. Cells were exposed to 8-Cl-cAMP (100 µM) or the PKA I-selective cAMP analogs (100 µM each) for 72 h and caspase 3/7 activity was determined with a luminescent assay. Data are from three independent experiments. * P<0.001 vs. control cells. All data are expressed as the variation versus control cell culture without drugs.

Next, we analyzed the release of histone-bound DNA fragments into the cytosol, as a marker of apoptotic DNA fragmentation. ARO, NPA and WRO cells were exposed to sub-maximal concentrations of the PKA I-selective cAMP analogs or 8-Cl-cAMP, and the amount of nucleosomes in the cytoplasm was evaluated by a specific ELISA. 8-Cl-cAMP treatment caused a significant increase in DNA fragmentation in all three cell lines that was detectable starting from 48 h of incubation. On the contrary, no induction of DNA fragmentation was observed in cells treated with the PKA I-selective cAMP analogs (Fig 2A and data not shown).

Finally, as apoptosis is associated with caspase activation [20], we examined the effect of both treatments on the activity of caspase 3/7, which was measured utilizing a luminescent assay. A significant increase in caspase 3/7 activity was detected in ARO, NPA and WRO cells exposed to 8-Cl-cAMP starting from 24 h of treatment, but not in the same cells treated with the PKA I-selective cAMP analogs (Figure 2B).

Taken together these data suggested that 8-Cl-cAMP but not the PKA I-selective cAMP analogs induced apoptosis in ARO, NPA and WRO cells.

### Apoptotic pathways activated by 8-Cl-cAMP

**Figure 3:**
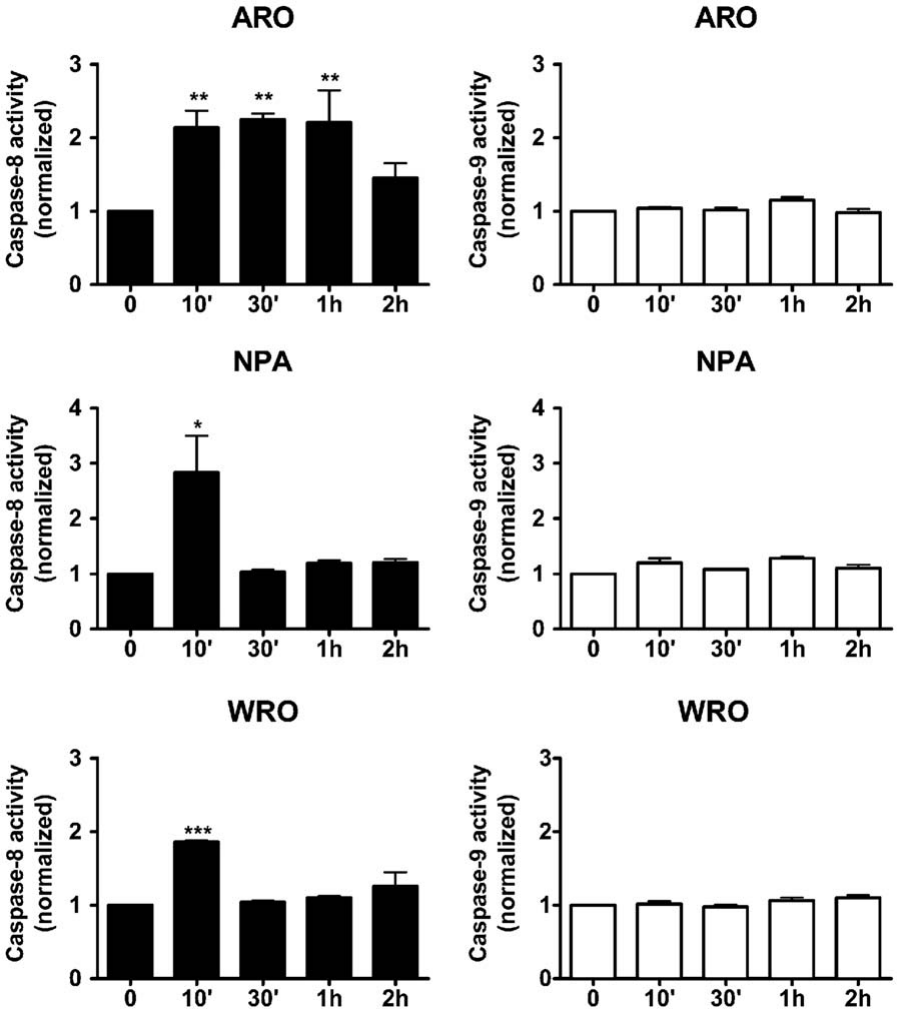
Effect of 8-Cl-cAMP on caspase 8 and 9. ARO, NPA and WRO cells were exposed to 8-Cl-cAMP (200 µM) for different periods of time and the activities of caspase 8 and 9 were measured utilizing specific luminescent assays. Data are from three to six independent experiments. * P<0.05 vs. basal. ** P<0.01 vs. basal. *** P<0.001 vs. basal. All data are expressed as the variation versus baseline.

To investigate the pathway involved in the apoptosis induced by 8-Cl-cAMP, we evaluated the activity of caspase 8 (extrinsic pathway) and caspase 9 (intrinsic pathway) utilizing specific luminescent assays. An early and transient (10 min–1 h) activation of caspase 8, but not of caspase 9, was observed (Figure 3), suggesting that 8-Cl-cAMP may induce the extrinsic pathway. However, these two caspases did not show any activation at later time points (24–48 h) (data not shown).

### Effect of 8-Cl-cAMP or the PKA I-selective cAMP analogs on the levels of PKA subunits

**Figure 4:**
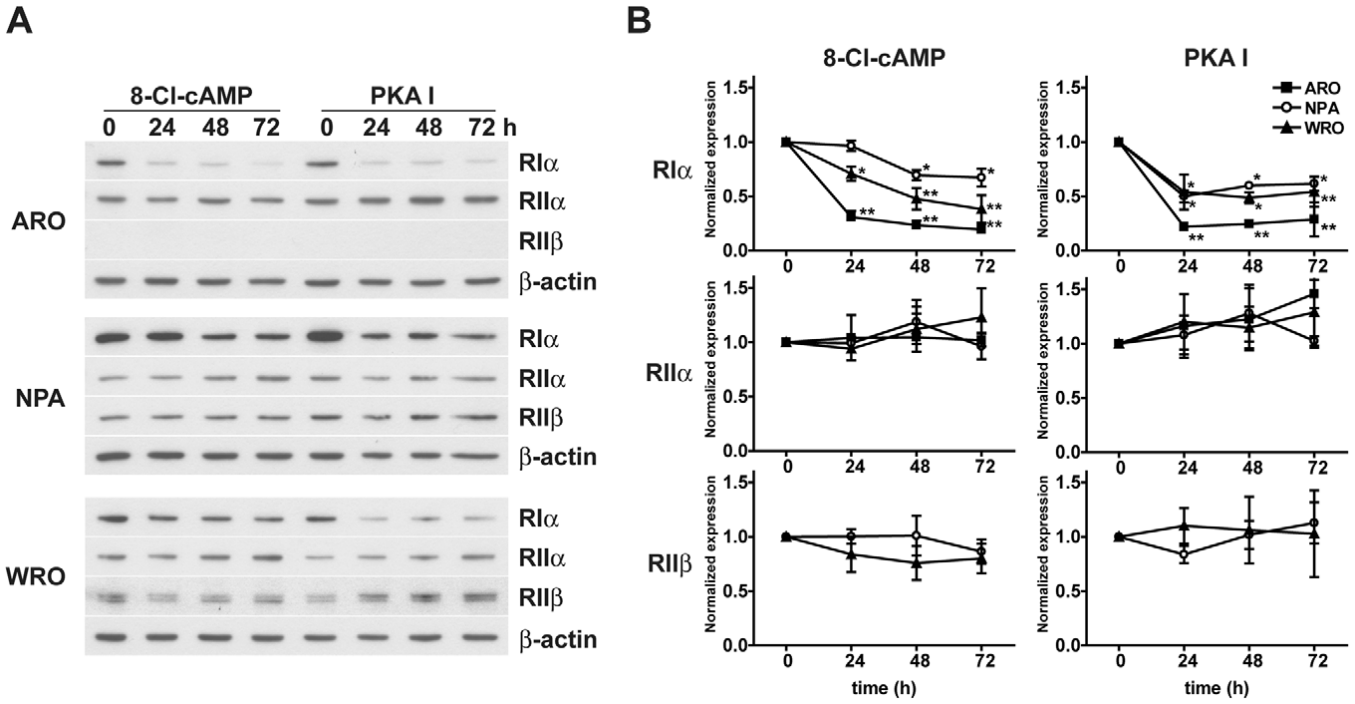
Effect of 8-Cl-cAMP or PKA I-selective cAMP analogs on the levels of PKA regulatory subunits. ARO, NPA and WRO cells were treated with 8-Cl-cAMP (100 µM) or the PKA I-selective cAMP analogs (100 µM each) for the indicated periods of time. The levels of RIα, RIIα and RIIβ were measured by Western blot analysis. Shown are representative Western blots (A) as well as the results of the densitometric analysis of four independent experiments (B). * P<0.01 vs. basal. ** P<0.001 vs. basal. These data are expressed as variation versus baseline.

Since it has been reported that 8-Cl-cAMP causes a down-regulation of RIα and an up-regulation of RIIβ subunits [14], [21], we compared the effects of 8-Cl-cAMP and the PKA I-selective cAMP analogs on the expression of the PKA R subunits. For this purpose, ARO, NPA and WRO cells were stimulated with 8-Cl-cAMP or the PKA I-selective cAMP analogs for different periods of time and the levels of RIα, RIIα and RIIβ were evaluated by Western blot analysis utilizing isoform-specific antibodies. Treatment of ARO, NPA and WRO cells with either 8-Cl-cAMP or the PKA I-selective cAMP analogs was associated with a reduction of the expression levels of the RIα subunit, whereas the levels of the remaining subunits were not affected (Figure 4).

### Effect of 8-Cl-cAMP or the PKA I-selective cAMP analogs on ERK 1/2 and Akt

In an effort to better understand the mechanism of action of 8-Cl-cAMP and the PKA I-selective cAMP analogs, we then examined the effect of both types of treatment on two key signaling cascades that are involved in cell growth, i.e. the extracellular signal-regulated kinase 1/2 (ERK) mitogen activated protein kinase (MAPK) pathway and the phosphatidylinositol 3-kinase (PI3K)/Akt pathway.

**Figure 5:**
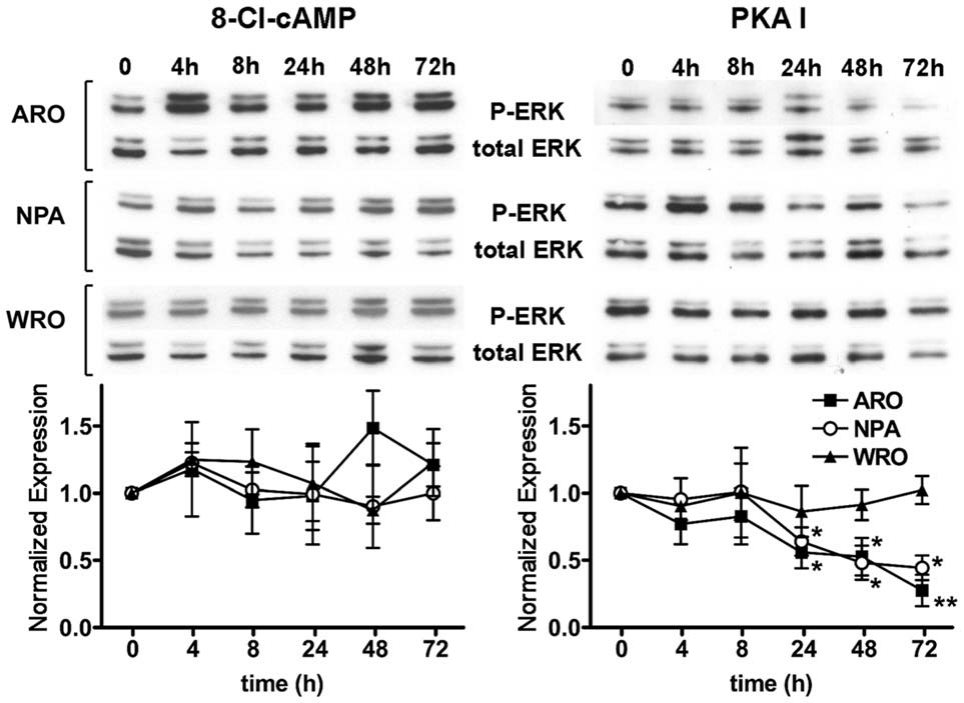
Effect of 8-Cl-cAMP or the PKA I-selective cAMP analogs on ERK phosphorylation. ARO, NPA and WRO cells were treated with 8-Cl-cAMP (100 µM) or the PKA I-selective cAMP analogs (100 µM each) for the indicated periods of time. The levels ERK 1/2 phosphorylated at Thr202/Tyr204 and total ERK were measured by Western blot analysis. Shown are representative Western blots as well as the densitometric analysis of three to four independent experiments. The P-ERK/total-ERK ratios are expressed as the relative variation versus the basal levels of each single experiment. * P<0.05 vs. basal. ** P<0.01 vs. basal.

ERK1/2 are well-characterized MAPKs that are activated in response to growth stimuli and play important roles in neoplastic transformation [22]. In addition, cAMP has been found to inhibit ERK 1/2 activation in several of the cell types where it has an antiproliferative effect [1], [2]. This seems to be the case also in ARO and NPA cells, where, as we have previously shown, the antiproliferative effect of the PKA I-selective cAMP analogs is associated with an inhibition of ERK 1/2 phosphorylation at Thr202/Tyr204 [19]. On this basis, we evaluated the effect of 8-Cl-cAMP treatment on ERK phosphorylation at Thr202/Tyr204 by Western blot analysis with a phospho-specific antibody. Analysis of ERK activation at early time points revealed transient increases for both treatments (Figure S3). At variance with what we previously observed in response to the PKA I-selective cAMP analogs, chronic exposure to 8-Cl-cAMP was not associated with a late and persistent inhibition of ERK phosphorylation (Figure 5).

Another protein that has been implicated in cell proliferation is the serine/threonine kinase Akt, which is activated by PI3K products and phosphorylation at Ser473 and Thr308 [23]. We therefore evaluated the effect of 8-Cl-cAMP and the PKA I-selective cAMP analogs on Akt phosphorylation at Ser473 and Thr308, indicative of its activation. Consistent with what we previously reported [19], treatment with 8-Cl-cAMP or the PKA I-selective analogs did not cause relevant modifications of Akt phosphorylation at early time-points (Figure S3) and for up to 72 h (data not shown).

### Effect of 8-Cl-cAMP or the PKA I-selective cAMP analogs on the p38 MAPK

**Figure 6:**
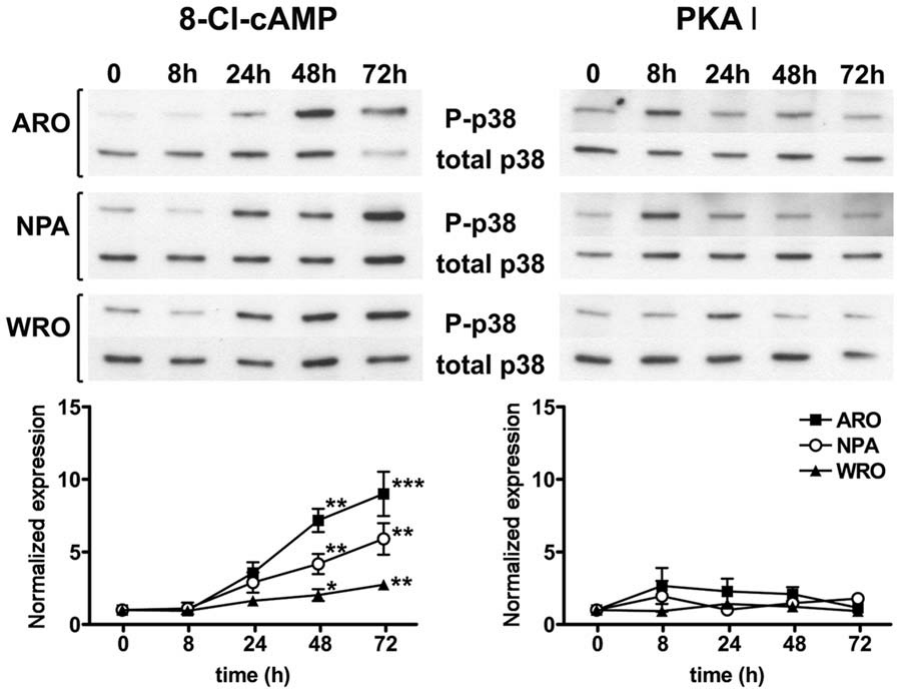
Effect of 8-Cl-cAMP on p38 MAPK phosphorylation. ARO, NPA and WRO cells were treated with 8-Cl-cAMP (100 µM) or the PKA I-selective cAMP analogs (100 µM each) for the indicated periods of time. The levels of phosphorylated and total p38 MAPK were measured by Western blot analysis. Shown are representative Western blots as well as the densitometric analysis of three independent experiments. P-p38/total-p38 ratio is normalized to basal levels. * P<0.05 vs. basal. ** P<0.01 vs. basal. *** P<0.001 vs. basal.

8-Cl-cAMP has been shown to induce p38 MAPK phosphorylation in HL60 and HeLa cells [24], [25]. Therefore, we evaluated the effect of 8-Cl-cAMP and the PKA I-selective cAMP analogs on p38 MAPK phosphorylation. We found that treatment of ARO, NPA and WRO cells with 8-Cl-cAMP was associated with a progressive increase in p38 MAPK phosphorylation, beginning at 24 h of incubation (Figure 6). On the contrary, treatment with the PKA I-selective cAMP analogs did not modify the phosphorylation state of the p38 MAPK (Fig 6), except for minor and transient increases that did not reach statistical significance. Moreover, treatment with 8-Cl-cAMP or the PKA I-selective analogs did not cause modifications of p38 MAPK phosphorylation at early time points (Figure S3).

**Figure 7:**
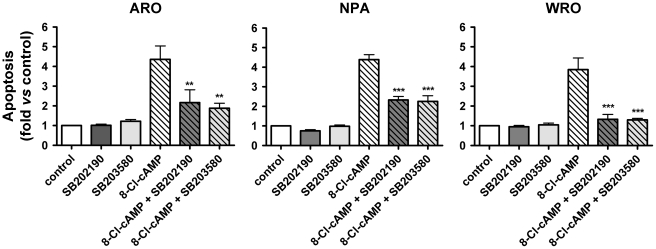
Effect of p38 MAPK inhibitors on the apoptosis induced by 8-Cl-cAMP. Cells were stimulated with 8-Cl-cAMP (100 µM) either in the presence or in the absence of p38 MAPK inhibitors (10 µM SB203580 or SB202190) for 72 h and apoptosis was evaluated by flow cytometry analysis after propidium iodide staining. Data are from at least three independent experiments. Data are expressed as the variation versus control cell culture. * P<0.05 and ** P<0.001 vs. cells treated with 8-Cl-cAMP alone.

Finally, we evaluated whether the pro-apoptotic effect of 8-Cl-cAMP was dependent on p38 MAPK activation. To this aim, we treated for 48–72 h all three cell lines with 8-Cl-cAMP in the presence or absence of selective p38 MAPK inhibitors (SB 202190 or SB203580) and analyzed the cell cycle distribution by flow cytometry as described above. Interestingly, inhibition of the p38 MAPK largely prevented the pro-apoptotic effect 8-Cl-cAMP (Figure 7).

Taken together, these data strongly suggest that the pro-apoptotic effect of 8-Cl-cAMP on ARO, NPA and WRO cells is mediated by the p38 MAPK.

### Involvement of AMPK in the activation of p38 MAPK by 8-Cl-cAMP

**Figure 8:**
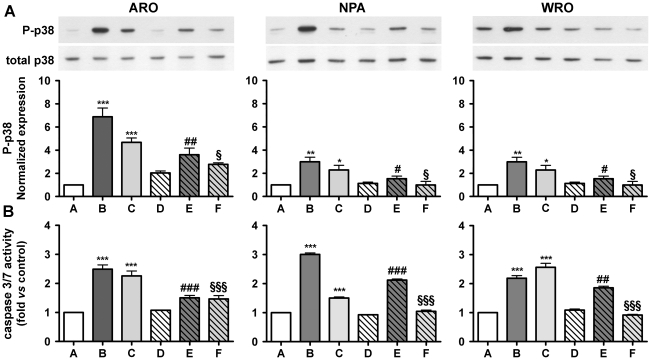
Involvement of AMPK in the activation of p38 MAPK and caspase 3/7 by 8-Cl-cAMP. ARO, NPA and WRO cells were stimulated with 8-Cl-cAMP (100 µM) or an AMPK activator (AICAR, 1 mM) for 72 h either in the presence or absence of pretreatment with an AMPK inhibitor (Compound C, 2.5 µM). A: the levels of phosphorylated and total p38 MAPK were measured by Western blot analysis. Shown are the results of representative Western blot experiments as well as the densitometric analysis of three independent experiments. Data are normalized to control levels. B: caspase 3/7 activity was determined with a luminescent assay. Shown are results from at least three independent experiments. Data are normalized to control cell culture. Legend: A: control, B: 8-Cl-cAMP, C: AICAR, D: Compound C, E: 8-Cl-cAMP + Compound C, F: AICAR + Compound C. * P<0.05, ** P<0.01, *** P<0.001 vs. control. # P<0.05, ## P<0.01, ### P<0.001 vs. 8-Cl-cAMP. § P<0.05, §§ P<0.01, §§§ P<0.001 vs. AICAR.

A recent study [25] has reported that the antiproliferative effects of 8-Cl-cAMP could be mediated by an activation of the AMP-activated protein kinase (AMPK) after its metabolization to 8-Cl-adenosine. To verify this hypothesis we evaluated the effects of both an activator, AICAR, and an inhibitor, Compound C, of AMPK. Treatment with AICAR mimicked the effects of 8-Cl-cAMP on p38 MAPK phosphorylation and on caspase 3/7 activity. In addition, Compound C was able to largely prevent both the phosphorylation of p38 MAPK and the activation of apoptosis induced by 8-Cl-cAMP (Figure 8).

## Discussion

cAMP analogs inhibit the proliferation of several tumor cells. The best studied of these compound is 8-Cl-cAMP, which has a demonstrated antiproliferative effect both *in vitro* and *in vivo* and has been evaluated in phase I/II clinical trials [11], [12], [13]. In a recent study, we have described the antiproliferative effect of a pair of cAMP analogs selective for PKA I on human carcinoma cell lines [19]. The goal of this work was to compare the effect of these cAMP analogs to that of the well studied 8-Cl-cAMP and further investigate their mechanism(s) of action. The results suggest that 8-Cl-cAMP and the PKA I-selective cAMP analogs, though of similar potency, differ in cell-type selectivity and mechanism(s) of action. The PKA I-selective cAMP analogs induce growth arrest, but not apoptosis, and, as we have previously shown, their effects appear to be mediated by a PKA-dependent inhibition of ERK activation. By contrast, 8-Cl-cAMP effects are most likely exerted by its metabolite 8-Cl-adenosine, are independent of PKA activation, and are apparently mediated by AMPK activation and subsequent p38 MAPK-dependent induction of apoptosis.

Our data indicate that 8-Cl-cAMP and the PKA I-selective cAMP analogs have a similarly potent antiproliferative effect, suggesting that both of them may retain a potential for cancer therapy. The PKA I-selective cAMP analogs seem to require the presence of constitutive ERK activation, as suggested by their being highly active only in the BRAF^V600E^-mutated ARO and NPA cells, where they inhibit ERK phosphorylation. By contrast, 8-Cl-cAMP strongly inhibits cell growth also in the BRAF-negative WRO cells, suggesting that it may be effective in a broader range of cancer cells.

Several findings indicate that 8-Cl-cAMP and the PKA I-selective cAMP analogs act via different mechanisms. First, the effect of 8-Cl-cAMP appears to be principally mediated, in agreement with previous observations [15], [16], [17], [18], by its metabolite 8-Cl-adenosine, acting via AMPK, and not by stimulation of PKA, as suggested by the effect of IBMX and of selective AMPK activation/inhibition; on the contrary, IBMX does not modify the antiproliferative response to the PKA I-selective cAMP analogs. Second, 8-Cl-cAMP and the PKA I-selective cAMP analogs produce dissimilar modifications of cell cycle. In particular, 8-Cl-cAMP, but not the PKA I-selective cAMP analogs, induce apoptosis. Third, the PKA I-selective cAMP analogs, but not 8-Cl-cAMP, reduce ERK phosphorylation in ARO and NPA cells. Fourth, 8Cl-cAMP, but not the PKA I-selective cAMP analogs, increase p38 MAPK phosphorylation.

The mechanism of action of 8-Cl-cAMP is highly debated. Cho-Chung and colleagues have done a considerable amount of work on this topic, the results of which indicate that 8-Cl-cAMP induces growth inhibition by increasing the RI to RII ratio [14], [21]. On the other hand, more recent studies suggest that this regulation is not the major cause of the observed growth inhibition [17]. In this report we find that 8-Cl-cAMP reduces the expression of RIα in ARO, NPA and WRO cells. However, we find that IBMX, which blocks the conversion of 8-Cl-cAMP to 8-Cl-adenosine, a metabolite unable to activate PKA, significantly reduces the antiproliferative effect of 8-Cl-cAMP.

A recent study [25] has demonstrated that 8-Cl-adenosine is able to stimulate AMPK, so we investigated the involvement of this pathway in 8-Cl-cAMP-induced apoptosis. Our data show that the induction of apoptosis by 8-Cl-cAMP is accompanied by the activation of caspase 3/7, and similar results were obtained by AICAR, an activator of AMPK. Furthermore, Compound C, an AMPK inhibitor, prevents the caspase 3/7 induction of both AICAR and 8-Cl-cAMP respectively. In the end, we propose that the pro-apoptotic effect of 8-Cl-cAMP is mediated by AMPK.

In addition, we show that the pro-apoptotic effect of 8-Cl-cAMP is accompanied by a progressive increase of p38 MAPK phosphorylation. The p38 MAPK is a key regulator of cell survival [26], [27], and has been implicated in the pro-apoptotic effect of 8-Cl-cAMP in HL60 and HeLa cells [24], [25]. To investigate if p38 MAPK activation is dependent by AMPK we employed an activation/inhibition strategy similar to that one adopted for caspase 3/7. Since AMPK blockade largely prevented and AMPK activation mimicked the effects of 8-Cl-cAMP on the p38 MAPK, our data strongly suggest that 8-Cl-cAMP after the conversion in its metabolite 8-Cl-adenosine activates the p38 MAPK through the stimulation of AMPK.

In an attempt to clarify the pathway leading to caspase 3/7 activation, we measured the activity of the upstream caspases 8 and 9, which are involved in the extrinsic and intrinsic apoptotic pathways, respectively. Whereas no activation of caspase 9 was observed, an early and only transient (5 min–1 h) activation of caspase 8 was detected. These data might indicate an involvement of the extrinsic pathway in the pro-apoptotic effects of 8-Cl-cAMP. However, given the observed long delay between the activation of caspase 8 and 3/7, and the transient nature of caspase 8 activation, it is possible that other mechanisms, likely independent of upstream caspases, might be involved in the activation of caspase 3/7. Interestingly, although the mechanisms by which the p38 MAPK can activate caspases are not fully elucidated, a possible direct activation of caspase 3 by the p38 MAPK has also been reported [28].

In conclusion, our data indicate that both 8-Cl-cAMP and the PKA I-selective cAMP analogs have a similarly potent antiproliferative effect on cancer cell lines of different origin. Thus, they both retain a potential as anticancer drugs. 8-Cl-cAMP appears to be effective in a broader range of cell types and induces apoptosis. On the other hand, the mechanism(s) of action of 8-Cl-cAMP and the PKA I-selective cAMP analogs are different, suggesting that they may have dissimilar pharmacological and toxicological profiles, as well as unique antitumoral properties. Future *in vivo* experiments will be required to compare their efficacy and tolerability in preclinical cancer models.

## Materials And Methods

### Chemicals

Cell culture reagents were purchased from Invitrogen (San Giuliano Milanese, Italy). 8-chloroadenosine-3′,5′-cyclic monophosphate (8-Cl-cAMP), 8-piperidinoadenosine-3′,5′-cyclic monophosphate (8-PIP-cAMP) and 8-hexylaminoadenosine-3′,5′cyclic monophosphate (8-HA-cAMP) were purchased from BIOLOG Life Science Institute (Bremen, Germany). The BCA kit and nitrocellulose membranes were purchased from Pierce Biotechnology (Rockford, IL). PKARIα, PKARIIα, PKARIIβ and β-actin antibodies were purchased from BD Biosciences Pharmingen (Milan, Italy). Phospho-ERK, phospho-Akt, phospho-p38 MAPK, ERK, Akt and p38 MAPK antibodies were purchased from Cell Signaling Technology (Beverly, MA). HRP conjugated mouse and rabbit secondary antibodies were purchased from Chemicon International (Temecula, CA). The ECL-plus kit was purchased from Amersham Biosciences Europe (Freiburg, Germany). The Cell Death Detection ELISA Plus kit was purchased from Roche Applied Science (Mannheim, Germany). The CellTiter-Blue cell viability and the Caspase-Glo 3/7, 8 or 9 assays were purchased from Promega Corporation (Madison, WI, USA). 4-(4-fluorophenyl)-2-(4-methylsulfinylphenyl)-5-(4-pyridyl)1H-imidazole (SB203580) was purchased from Biosource (Nivelles, Belgium). 3-isobutyl-1-methylxanthine (IBMX), 4-(4-fluorophenyl)-2-(4-hydroxyphenyl)-5-(4-pyridyl)-1H-imidazole (SB202190), bisindolyl-maleimide I (GF109203X), AICAR, 6-[4-(2-Piperidin-1-ylethoxy)phenyl]-3-pyridin-4-ylpyrazolo[1,5-a]pyriimdine (Compound C) and all other reagents were purchased from Sigma-Aldrich (Milan, Italy).

### Cell culture

ARO, NPA and WRO cells were kindly provided by I. Bongarzone (Milan, Italy). A recent DNA profiling analysis has revealed that ARO cells match the HT-29 colon cancer cell line and NPA cells match the M14/MDA-MB-435S melanoma cell line [29]. WRO cells derive from a follicular thyroid carcinoma [30]. All cells were maintained in DMEM medium supplemented with 10% FCS, 1% penicillin and 1% streptomycin at 37°C in a humidified atmosphere with 5% CO_2_.

### Selective activation of PKA I

To selectively activate PKA I, we utilized the commonly used strategy of simultaneously utilizing two distinct cAMP analogs, i.e. 8-piperidinoadenosine-3′,5′-cyclic monophosphate (8-PIP-cAMP) and 8-hexylaminoadenosine-3′,5′cyclic monophosphate (8-HA-cAMP), each with high selectivity for binding to either site A (8-PIP-cAMP) or B (8-HA-cAMP) of type I PKA R subunits [19].

### Proliferation assay

Cell proliferation was evaluated utilizing the 3-(4,5-dimetylthiazole-2-yl)-2,5-diphenyltetrazolium bromide (MTT) assay, as previously described [19]. Briefly, cells were seeded in 96-well plates, and 24 h after plating were treated with the indicated concentrations of 8-Cl-cAMP or the PKA I-selective cAMP analogs (8-PIP-cAMP + 8-HA-cAMP). For inhibition of PKC, cells were incubated for 30 min with GF109203x prior to addition of 8-Cl-cAMP or the PKA I-selective cAMP analogs. MTT (0.5 mg/mL) was added to cells three hours before measurement, and formazan crystals, formed by mitochondrial reduction of MTT, were solubilized in DMSO/ethanol 1∶1. Relative growth was calculated from the absorbance at 550 nm utilizing the following equation: Relative growth (%) = (OD_550nm_ of treated wells/OD_550nm_ of control wells) ×100.

### Detection of apoptotic DNA fragmentation

DNA fragmentation was evaluated utilizing the Cell Death Detection ELISA Plus kit. This assay provides a quantitative determination of histone-associated DNA fragments (mono- and oligo-nucleosomes) in the cytoplasmatic fraction of cell lysates. Briefly, cells were seeded in 96-well plates and 24 h later treated with 8-Cl-cAMP or the PKA I-selective cAMP analogs for 48 h. The samples were then processed and analyzed in triplicate, according to the manufacturer's instructions.

### Determination of caspase activities

Caspase activity was measured utilizing the luminescent Caspase-Glo 3/7, 8 or 9 assays, following the manufacturer's instructions. Caspase 3/7 activity was normalized for the number of cells contained in each well, determined utilizing the fluorometric CellTiter-Blue cell viability assay. Activation levels of caspases 8 and 9 are expressed relative to non-treated samples. Luminescent and fluorescent measurements were performed utilizing the Fluoroskan Ascent FL multiplate reader (Thermo Labsystems, Helsinki, Finland).

### Analysis of cell cycle by flow cytometry

The cells were harvested by trypsinization, washed in PBS and then fixed with ice-cold 70% ethanol. Fixed cells were washed with PBS and stained with PBS containing 40 µg/ml propidium iodide and 100 µg/ml RNAse A at 37°C for 30 min. Samples were analyzed with the FACSCalibur flow cytometer (Becton Dickinson, Buccinasco, Italy).

### Western blot analysis

For detection of PKA regulatory subunits, cells were lysed in modified RIPA buffer containing protease inhibitors (10 mM Tris-HCl pH 7.5, 500 mM NaCl, 0.1% SDS, 1% NP40, 1% Na deoxycholate, 2 mM EDTA, 2 mM Na_2_VO_4_, 2 mM Na_4_P_2_O_7_, 2 mM NaF). For detection of ERK, Akt, and p38 MAPK phosphorylation, cells were lysed with SDS sample buffer (62.5 mM Tris-HCl pH 6.8, 2% SDS, 10% Glycerol, 50 mM DTT, 0.01% Bromophenol Blue), immediately heated for 5 min at 95°C and sonicated. Protein concentration was determined utilizing the BCA assay. Ten micrograms of protein extracts were separated on 10% SDS polyacrylamide gels and electrotransfered to nitrocellulose membranes. Membranes were blocked with TBS-T +5% milk, probed with the indicated primary antibody overnight at 4°C and incubated with an appropriate HRP-conjugated secondary antibody for 1 h at room temperature. The detection was performed utilizing the ECL-plus kit.

### Statistical analysis

Values are expressed as mean ± standard error. One-way or two-way ANOVA followed by Bonferroni's post-hoc test were used, as appropriate, to evaluate differences between means. A P value <0.05 was considered statistically significant.

## Supporting Information

Figure S1 **Effect of phosphodiesterase inhibition.** Cells were preincubated with IBMX (50 µM) for 1 h before addition of 8-Cl-cAMP (100 µM) or the PKA I-selective cAMP analogs (100 µM each) for 72 h. Cell viability was determined utilizing the MTT assay. * P<0.001 vs. control. ** P<0.05 vs. control. § P<0.001 vs. cells treated with 8-Cl-cAMP alone.
(TIF)

Figure S2 **Effect of PKC inhibition.** Cells were preincubated with a PKC inhibitor (GF109203x, 1 µM) for 1 h before addition of 8-Cl-cAMP (100 µM) for 72 h. Cell viability was determined utilizing the MTT assay.
(TIF)

Figure S3 **Early effects of 8-Cl-cAMP and PKA I-selective cAMP analogs on ERK, p38 MAPK and Akt phosphorylation.** Shown are the results of the densitometric analysis of three independent Western blot experiments per condition. Data are normalized to basal levels. Differences between peak and basal values of ERK phosphorylation are statistically significant in ARO, WRO and NPA cells after stimulation with both 8-Cl-cAMP (P<0.05, P<0.01 and P<0.05, respectively) and PKA I-selective analogs (P<0.001, P<0.05 and P<0.05, respectively). Differences of Akt and p38 MAPK phosphorylation are not statistically significant.
(TIF)
