# Supplementary figures and images for: 8-Chloro-Cyclic AMP and Protein Kinase A I-Selective Cyclic AMP Analogs Inhibit Cancer Cell Growth through Different Mechanisms
Source: PLoS One. 2011 Jun 10;6(6):e20785. doi: 10.1371/journal.pone.0020785 (PMC3112188; doi:10.1371/journal.pone.0020785)

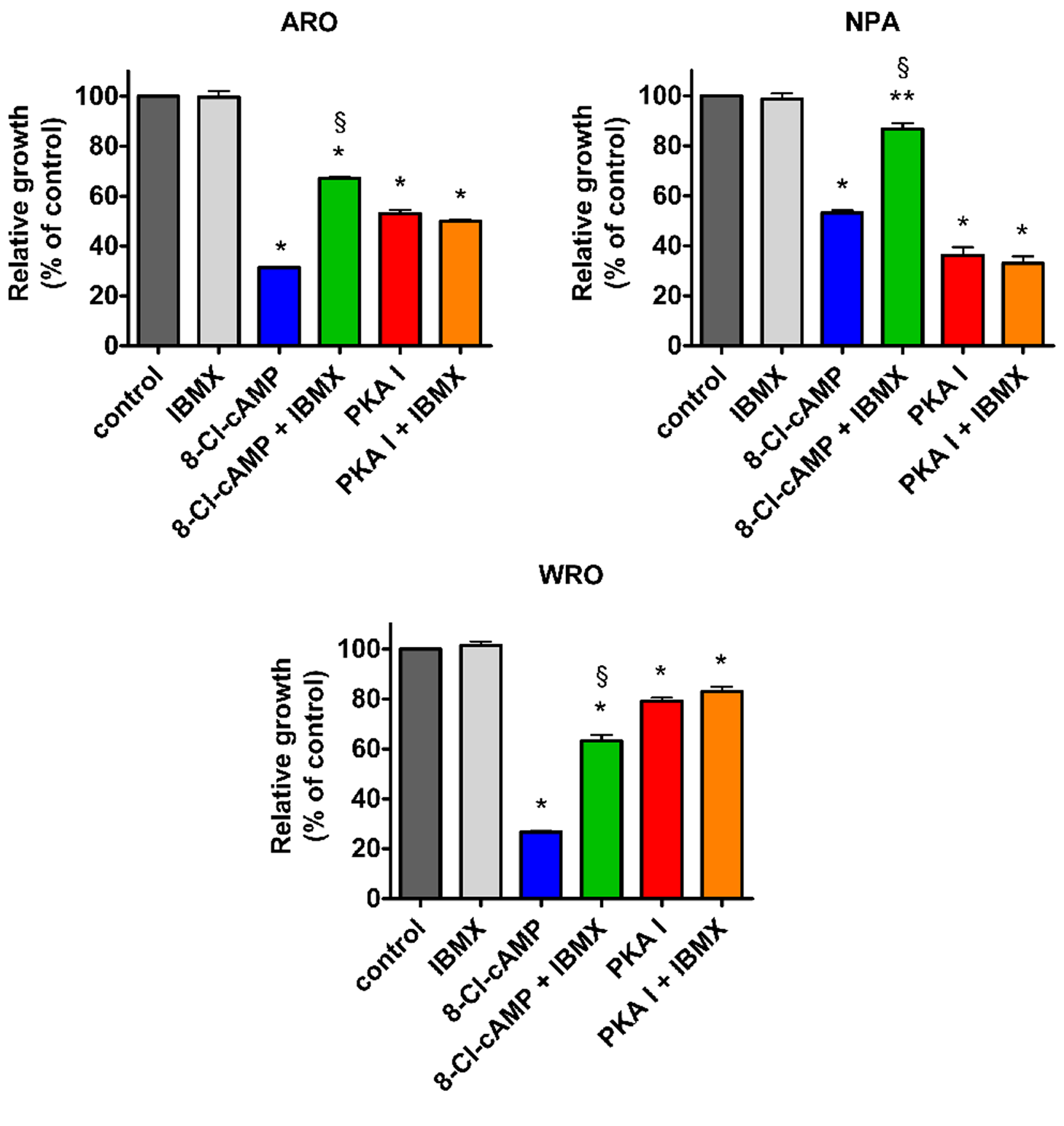

Supplement: Figure S1 — Effect of phosphodiesterase inhibition. Cells were preincubated with IBMX (50 µM) for 1 h before addition of 8-Cl-cAMP (100 µM) or the PKA I-selective cAMP analogs (100 µM each) for 72 h. Cell viability was determined utilizing the MTT assay. * P<0.001 vs. control. ** P<0.05 vs. control. § P<0.001 vs. cells treated with 8-Cl-cAMP alone. (TIF) [file pone.0020785.s001.tif]

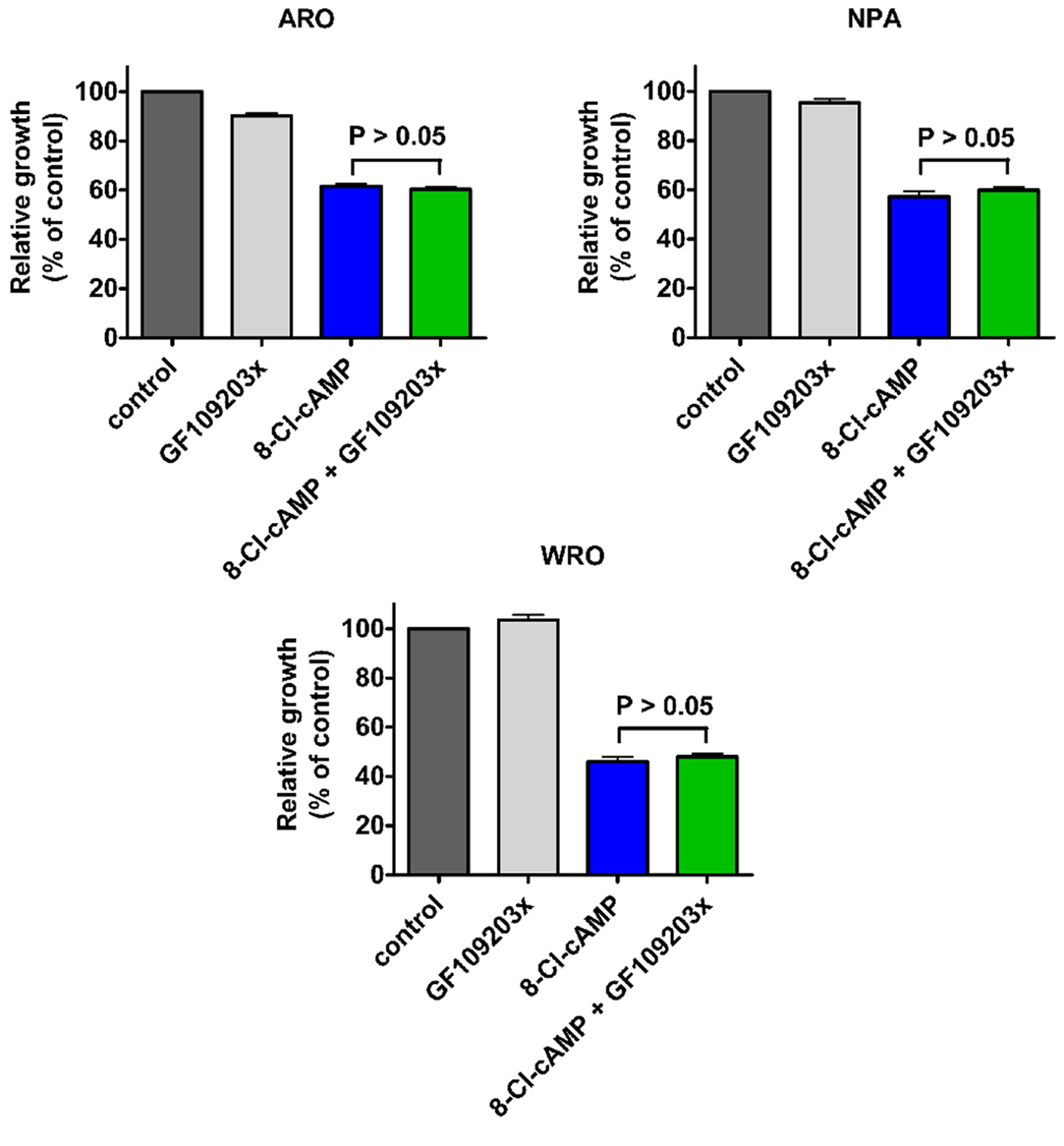

Supplement: Figure S2 — Effect of PKC inhibition. Cells were preincubated with a PKC inhibitor (GF109203x, 1 µM) for 1 h before addition of 8-Cl-cAMP (100 µM) for 72 h. Cell viability was determined utilizing the MTT assay. (TIF) [file pone.0020785.s002.tif]

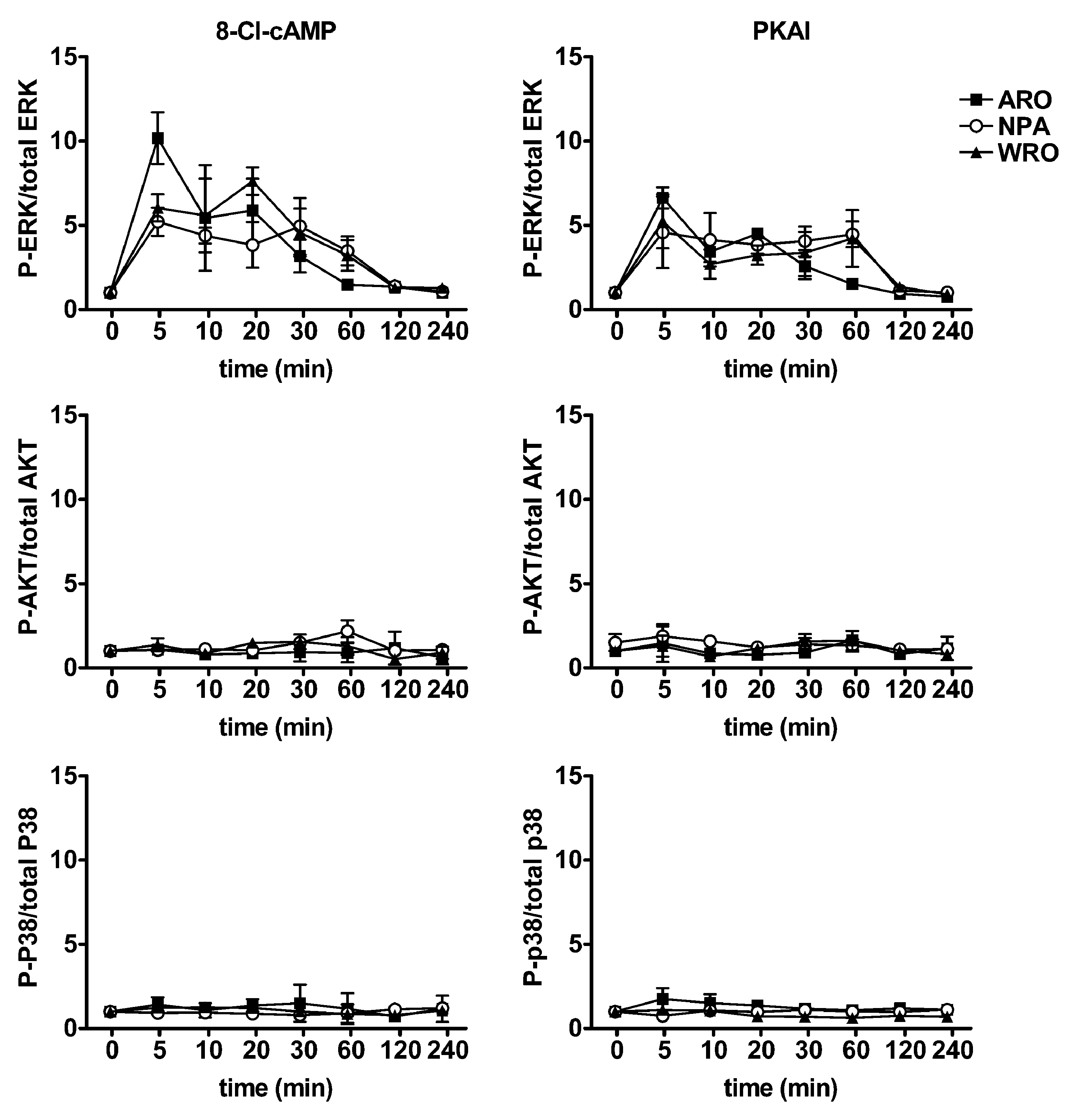

Supplement: Figure S3 — Early effects of 8-Cl-cAMP and PKA I-selective cAMP analogs on ERK, p38 MAPK and Akt phosphorylation. Shown are the results of the densitometric analysis of three independent Western blot experiments per condition. Data are normalized to basal levels. Differences between peak and basal values of ERK phosphorylation are statistically significant in ARO, WRO and NPA cells after stimulation with both 8-Cl-cAMP (P<0.05, P<0.01 and P<0.05, respectively) and PKA I-selective analogs (P<0.001, P<0.05 and P<0.05, respectively). Differences of Akt and p38 MAPK phosphorylation are not statistically significant. (TIF) [file pone.0020785.s003.tif]
